# Relations among Pro-Environmental Behavior, Environmental Knowledge, Environmental Perception, and Post-Materialistic Values in China

**DOI:** 10.3390/ijerph19010537

**Published:** 2022-01-04

**Authors:** Jinchen Xie, Chuntian Lu

**Affiliations:** Department of Sociology, Xi’an Jiaotong University, Xi’an 710049, China; jinchenxie@stu.xjtu.edu.cn

**Keywords:** pro-environmental behavior, environmental knowledge, environmental perception, post-materialistic values

## Abstract

During the economic boom, China’s government was mainly concerned with economic development; however, numerous environmental problems have arisen. Evidence suggests that Chinese individuals’ pro-environmental behavior (PEB) is at a low level in Asia. However, it does not match their high-quality environmental knowledge. In this paper, the database of the Chinese General Social Survey was used to explore the correlation between environmental knowledge and PEB in a broader context. Subsequently, environmental perception and post-materialistic values (PMV) were taken as the mediator and moderator into structural equation modeling, and every variable kept robust and consistent through exploratory factor analysis. The empirical results indicated that: (i) individuals with higher environmental knowledge always show higher passion to PEB; (ii) environmental perception plays a partially mediating role between environmental knowledge and PEB; (iii) PMV moderate the formation of environmental behavior systematically; and (iv) compared with public counterpart, the relation between environmental knowledge and PEB is significantly higher in private environmental behavior. The study results could become the basis for the Chinese government and environmental NGOs to effectively spread environmental knowledge, advocate a post-materialistic lifestyle, and improve the authenticity of online media reports on environmental issues.

## 1. Introduction

With the rapid development of China’s economy and the continuous advancement of urbanization [1], a series of environmental problems are becoming increasingly worse, including the greenhouse effect, water pollution, acid rain, and desertification [2]. This is attributed to the decline in Chinese pro-environmental behavior (PEB). Recently, evidence has suggested that Chinese residents’ PEB is nearly the last among Asians, which is inconsistent with their economic situation [3]. However, according to several large-scale comprehensive surveys, Chinese individuals’ environmental knowledge is at the highest level in the world [4]. In other words, Chinese individuals’ environmental knowledge has not been effectively transformed into PEB, and this needs to be paid special attention.

Recently, abundant literature has tried to fill this correlation gap between environmental knowledge and environmental behavior, and found that environmental knowledge affects PEB mainly through two systematic methods [5]. On the one hand, according to the theory of planned behavior, environmental knowledge can directly stimulate environmental behavior [6], and this viewpoint is supported by abundant of literature. Furthermore, researchers have found that this positive effect exists in East Asia, the United States, and Europe, simultaneously. For a specific country, the relevance between environmental knowledge and environmental behavior might change with individual demographic characteristics. More specific, compared with men and the poor, women and the rich are more likely to transform environmental knowledge into environmental behavior.

On the other hand, environmental knowledge indirectly affects PEB through some mediating factors. This viewpoint attracts extensive attention of researchers. For example, the typical normal activation model indicated that pro-environmental conceptual schema played a mediating role between the environmental knowledge and PEB [7]. Meanwhile, environmental perception can significantly explain the indirect effect between environmental knowledge and PEB, especially in Europe and the United States [8,9,10]. However, it should be pointed out that almost all researchers ignore that environmental perception can be divided into different categories, which lead to the heterogeneous mediating effects. In addition, some Chinese researchers believe that the media usage is a potential mediating variable between environmental knowledge and PEB [11]. Although, more researchers used environmental knowledge as a control variable in their empirical research.

However, based on the above analysis, with respect to the Chinese context, a fundamental factor must be taken into consideration, namely, post-materialist values. Because Chinese individuals are always regarded as the following contradictory characteristics: rich but lacking environmental morality. However, a few studies have mentioned that post-materialistic values (PMV) may moderate the relationship between environmental knowledge and PEB among Chinese individuals. In addition, whether or not the mediating effects of environmental perception is still significant in China is worth discussing, because environmental perception usually arises from media rather than direct perception. However, China’s media tend to cover up the truth of environmental issues for economic purposes. Additionally, a few studies have noticed that environmental behavior and environmental knowledge contain different types [12,13]. For example, environmental knowledge about water pollution may have a positive impact on public environmental behavior, but not on private behavior. Therefore, this paper tried to set up systematic correlations between environmental knowledge and PEB, and took environmental perception and PMV as the mediator and/or moderator, respectively.

## 2. Basic Concepts: Pro-Environmental Behavior, Environmental Knowledge, Environmental Perception, Post-Materialistic Values

At present, China’s government is confronted with the challenge of greenhouse gas emission reduction, which is highly connected with the reverse of the environmentally unfriendly lifestyles. At the same time, China has become the largest garbage producer and greenhouse gas emitter country in the word since about 2005 [14]. Chinese residents’ perception of environmental pollution in their daily life is much higher than the international average level. In addition, as the second largest economic entity, China has achieved modernization as a whole. Therefore, it is very important to investigate the impact of PMV on environmental behavior. Furthermore, in order to accurately obtain the relevant mechanism, it is of vital necessity to focus on the correlation between environmental knowledge and PEB with the moderating or mediating of environmental perception and PMV.

Pro-environmental behavior (PEB): PEB is usually defined as behavior that individuals spontaneously undertake in their daily life and contribute to the improvement of environmental quality. In this study, PEB was divided into public PEB and private PEB, and they are conceptually distinct. Private PEB can be conceptualized as the PEB that is generated from an individuals’ daily life, such as recycling plastic bags. Public PEB is the process by which individuals participate in public environmental protection in response to the call of the government or social organizations, such as participating in environmental debates.

Environmental knowledge: Environmental knowledge refers to the cognition of the environmental problems, environmental science, and environmental governance. Generally speaking, environmental knowledge is divided into daily environmental knowledge and professional environmental knowledge. Daily environmental knowledge can be conceptualized as the environmental knowledge related to the common environmental pollution in life, such as harmful noise. Professional environmental knowledge usually occurs in specialized fields (e.g., chemical pharmacy, industrial production, and soil research), which are little known to the public, such as a single species of forest being susceptible to diseases.

Environmental perception: Environmental perception is conceptualized as the subjective judgment and direct feeling of the individuals facing objective environmental risks, such as an environmental phenomenon directly perceived by the residents, which will have a significant impact on environmental protection behavior. Capaldi et al. (2014) divided environmental perception into two systematically and different kinds: ecological environmental perception and daily environmental perception [15]. An individuals’ perception of all natural, social, or cultural environments of human life is usually defined as ecological environmental perception, and the perception of daily environmental problems is defined as daily environmental perception.

Post-materialistic values (PMV): PMV can be defined as the cultural concepts of social groups, which get rid of the “materialism” that focuses on meeting basic survival needs, and turns to the pursuit of high-quality life security. Ronald and Inglehart (1995) believed that after the material needs are met, post-materialistic behaviors such as environmental protection, democratic consciousness, political participation, and gender equality will systematically appear in society [16].

The existing literature rarely considers the above four factors in a specific model, especially where this relationship is positioned in a broader context of institutions and their effects. Thus, we took environmental perception as a mediator because the existing literature proved that environmental knowledge could stimulate environmental perception, and environmental perception will affect environmental behavior. Meanwhile, we took PMV as a moderator, to investigate whether PMV can positively impact the generation of residents’ PEB. The added value of our research is threefold. First, PMV was taken into our research, and answered whether it played a part in the formation of Chinese PEB; second, we classified environmental knowledge, PEB, and environmental perception into different types, and discussed the correlation among them, respectively; third, we tried to position the correlation between these four factors into a specific model, so as to construct a moderated mediating model and further discussion. Appropriate research questions were formulated as below:

1. For Chinese residents, is there a significant relationship between environmental knowledge and PEB? Is the relationship between different types of environmental knowledge and behavior consistent?
2. Does environmental perception play a role in the above process as a mediator? Is the mediating effect complete or partial?
3. Does PMV participate in the above process as a moderator? Is the moderating effect positive or negative?

## 3. Methodology

### 3.1. Data

We collected data from the Chinese General Social Survey (CGSS), which was jointly conducted by Renmin University of China and relevant academic institutions. The respondents were adults in Mainland China, including over 10,000 samples from 31 provinces/municipalities in China. Since the first round of survey in 2003, CGSS had completed ten rounds of annual survey, which fully reveals the characteristics of Chinese individuals. It is worth noting that the focus of each round of CGSS is significantly different, just like the World Value Survey. The environmental issues concerned in this paper are the main focus of CGSS in 2013. Therefore, we chose the survey data in CGSS in 2013. After dropping the invalid respondents, the final sample size was 11,438.

### 3.2. Measures

#### 3.2.1. Dependent Variable

PEB is the dependent variable to which we paid attention. Exploratory factor analysis (EFA) was conducted on ten related PEBs of CGSS in 2013. Two common factors, public PEB and private PEB, were obtained (KMO = 0.826, Bartlett < 0.001). The private PEB contains (a) garbage classification, (b) rarely use plastic bags, (c) pay attention to environmental news, and (d) discuss environmental issues with others. The public PEB contains (a) donate for environmental protection, (b) actively participate in public environmental affairs, (c) actively participate in environmental NGOs, (d) protect trees or green space spontaneously, and (e) complaints for environmental protection. (KMO = 0.826, Bartlett < 0.001) PEB was coded 1–3 as “never participate”, “generally” and “always participate”.

#### 3.2.2. Independent Variable

Environmental knowledge is the main independent variable in our research. The conceptual framework of Bradley and Waliczek (1999) was referred to extract variables from CGSS in 2013. After conducting EFA, two common factors were extracted. The former contains three elements: (a) overuse of chemical fertilizer is harmful, (b) fluorine destroys the ozone layer, and (c) using coal leads to acid rain. The latter contains: (a) every species is important in the biological chain, (b) a single species of forest is susceptible to diseases, and (c) carbon dioxide causes air warming. (KMO = 0.816, Bartlett < 0.001). It should be noted that the former three factors are generally recognized in China, while the latter three factors are less understood. Thus, we conceptualized these two factors as general environmental knowledge and professional environmental knowledge, respectively. Every variable was assessed by a binary scale (0 = no 1 = yes), each question has a standard answer, and the data we used were also expressed by binary variables, 0 and 1 denotes wrong or right, respectively.

#### 3.2.3. Mediating Variable

According to theoretical analysis, environmental perception was taken as a mediating variable among the correlation of different environmental knowledge and different PEB. We conducted EFA on the twelve related factors of environmental perception in CGSS in 2013, and finally extracted two common factors: daily environmental perception and ecological environment perception. The former contains the following seven elements: (a) air pollution, (b) water pollution, (c) noise, (d) industrial garbage, (e) daily garbage, (f) lack of green space, and (g) food pollution. The latter contains: (a) forest destruction, (b) cultivated land degradation, (c) lack of fresh water, (d) desertification, and (e) reduction in wildlife (KMO = 0.913, Bartlett < 0.001). All factors were rated as the 5-point Likert scale (1 = lightly perceive; 5 = strongly perceive).

#### 3.2.4. Moderating Variable

We focused on the process that PMV shapes the PEB of Chinese residents; thus, we took PMV as the moderating variable. EFA was conducted to extract five common factors representing PMV from CGSS in 2013, which included (a) government needs democracy, (b) the people must have a voice of public affairs, (c) representatives should express public opinion, (d) people’s benefits are more important than the national, and (e) people should participate in national decision-making (KMO = 0.801, Bartlett < 0.001). All factors were binary (0 = disagree and 1 = agree), and we added them up and obtained a continuous variable, a higher score meant more positive in post materialist values.

### 3.3. Analysis

Structural Equation Modeling (SEM) has been widely applied to estimate the structural correlations among the latent variables. SEM was chosen in this study because of the following two reasons. First, we paid more attention to the correlation among latent variables, which are difficult to achieve in the traditional regression method. While SEM is an appropriate analytical approach to discuss the correlation among latent variables. Second, we constructed a mediation model, and SEM is more effective to reveal the relationship among variables in the complex model. The following procedures were conducted in our research: (i) the EFA of four latent variables was firstly conducted, including PEB, environmental knowledge, environmental perception, and PMV; (ii) descriptive analysis of all variables was reported; (iii) SEM was conducted to identify the correlation among the environmental knowledge and PEB, describing the correlation of between these two factors preliminarily; and (iv) mediator and moderator were added to draw the systematically correlations between these four factors.

## 4. Results

### 4.1. Descriptive Statistics

The comprehensive information of the whole variables (e.g., correlations, mean, and standard deviation) are listed, as shown in Table 1. The correlations between each adjacent variable are statistically significant, except the correlation between materialistic-value and public PEB, which indicated that our data highly support the subsequent SEM model. The normality assumption of all the variables were well met, where the skewness values were less than 3 and kurtosis values were less than 10.

### 4.2. SEM Model

**Figure 1:**
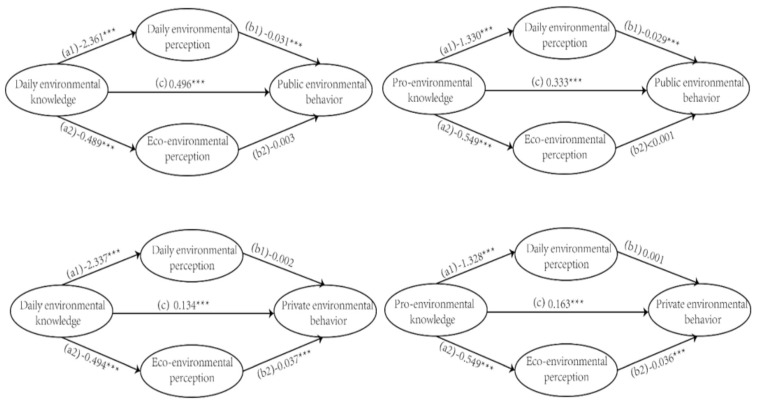
Final model estimations with standardized beta weight (SE). Note. *** *p* < 0.05.

The whole model was highly acceptable (chi square = 3170.97, CFI = 0.924, TLI = 0.879, RMSEA = 0.057, 90% C.I. = (0.056, 0.058)). To display the outcome clearly, the model was divided into four groups, respectively(shown as Figure 1). Firstly, we focused on the correlation between daily environmental knowledge and public environmental behavior, the direct coefficient of these two factors was positive and significant (β = 0.496, *p* < 0.001). The result also showed that the indirect relationship between these two factors via daily environmental perception and ecological environment perception was significant (indirect effect = 0.074, 95% CI (0.054, 0.095)) and significant (indirect effect = 0.002, 95% CI (−0.003, 0.006)), respectively. Our result indicated that the daily environmental knowledge of Chinese residents can effectively stimulate the generation of public PEB. For example, when Chinese residents have understood that automobile exhaust emissions are harmful to health (Daily environmental knowledge), they would call for public transport instead of driving (Public environmental behavior). Additionally, the daily environment perception partially mediates the above process. Such conclusion is consistent with the extent literature [17,18].

Secondly, the net effects between professional environmental knowledge and public PEB was identified, and the coefficient was significantly positive (β = 0.333, *p* < 0.001). The indirect relationship between these two factors through daily/ecological environmental perception was significant (indirect effect = 0.039, 95% CI (0.029; 0.050)) and non-significant (indirect effect < 0.001, 95% CI (−0.004; 0.004)), respectively. Compared with daily environmental knowledge, professional environmental knowledge showed systematically disadvantage in stimulating public PEB, whether directly or indirectly. These conclusions are rarely found in previous literature because the previous studies rarely divided environmental behavior and environmental knowledge into different types nor discussed the relationship between them in-depth [19,20].

Thirdly, the correlation of daily environmental knowledge and private PEB was positive statistically (β = 0.15, *p* < 0.001). It should be pointed out that the indirect correlation is totally different from the mentioned above. Compared with daily counterpart (indirect effect < 0.001, 95% CI (−0.004, 0.004)), the ecological environment perception significantly mediated the relationship between daily environmental knowledge and private PEB (indirect effect = 0.039, 95% CI (0.029, 0.050)). More specific, Chinese residents may be more concerned about the public environmental crisis, rather than pollution in daily life. This conclusion is inconsistent with the literature describing the characteristics of the Chinese as lacking public morality [21,22]. The reason is that in China, individual benefits are far less important than public benefits.

Fourth, a positive correlation between professional environmental knowledge and private PEB was obtained (β = 0.16, *p* < 0.001). Comprehensively speaking, environmental knowledge was better at stimulating the generation of public PEB (the direct relation in model 1,2) rather than private PEB (the direct relation in model 3,4). This also supports the conclusion that we refuted the Chinese people’s lack of public morality in the previous paragraph. Meanwhile, the indirect correlation between these two factors through daily/ecological environment perception showed non-significant (indirect effect = −0.001, 95% CI (−0.008, 0.007)) and significant (indirect effect = 0.020, 95% CI (0.013, 0.027)), respectively. 

Lastly, as suggested in theoretical analysis, the PMV may moderating the formation of PEB. So, we added the PMV into SEM, and the moderating effects were obtained, as shown in Table 2. A panoramically positive moderating effects indicated that PMV was systematically benefit for the formation of PEB, whether through environmental knowledge or environmental perception. The results showed that when people’s values shift from materialism to post-materialism, Chinese residents’ PEB will be systematically improved, as shown in Table 3.

## 5. Discussion

The results indicated that daily/professional environmental knowledge positively predicts the formation of public PEB, which means that enhancing Chinese residents’ environmental knowledge will significantly improve their public PEB. Meanwhile, the indirect correlation among these three factors through environmental perception showed significantly positive consistence, except that ecological environment perception partially mediated the formation of PEB. Such results revealed that environmental perception (whether public or ecological) plays a part of active mediation in the process of transforming environmental knowledge into public PEB.

It is worth mentioning that the private PEB has a comparatively mild positive relationship between environmental knowledge and environmental perception compared with the public counterpart. The result is consistent with conventional view that Chinese individuals pay more attention to the cleanliness of their private environment and lack public awareness of environmental protection. The direct correlation among daily/professional environmental knowledge and private PEB significantly positive, respectively. Based on the above conclusions, if we want to change the current situation of weak PEB of Chinese residents, it is of vital necessity to increase environmental knowledge (whether daily or professional). The indirect correlation among these three factors through environmental perception showed systematically insignificant, except the mediating of ecological environment perception in the correlation between professional environmental knowledge and private PEB. Thus, we conclude that daily/ecological environmental perception is more likely to mediate the formation of pubic PEB than private PEB.

Of interest, such conclusions are consistent with several extent literature about Chinese individuals’ PEB, which indicated a positive correlation between environmental knowledge and PEB [23,24]. However, Chinese residents are systematically insensitive to the mediating role of environmental perception in this correlation. While several European and American researchers found that environmental perception significantly mediated the generation of PEB [25,26]. Our result showed that Chinese individuals may be less sensitive to environmental issues.

As for the moderating effects of PMV into the formation of PEB, Chinese residents showed statistically positive significance. Our results indicated that it is still applicable for the PMV theory in China context, which is in line with the content literature [27,28]. Data showed that there is a mismatch between the lack of PEB and the high-quality economic of China. According to our empirical analysis, when people with materialistic values are gradually replaced by people with PMV, Chinese residents’ PEB will be systematically improved.

In summary, environmental knowledge and PMV are attributed to the formation of Chinese residents’ PEB significantly, while environmental perception played a partial role. A conceivable explanation for the outcomes is that Chinese residents are insensitive to environmental issues. Generally speaking, individual environmental perception is obtained mainly from network media rather than daily experience. However, China’s media have been criticized for a long time, and they usually hide the truth of environmental problems for commercial purposes. Therefore, the environmental perception of Chinese residents is generally not as abundant as it should be, and it is difficult for environmental perception to participate in the formation of PEB. The above conclusions also inspire researchers to pay more attention to the correlation between network media, environmental perception, and PEB, especially in Western countries.

In the context of policy, these results underpin several supporting to ongoing environmental investments in China. During the economic boom, China’s government has paid the majority of its attention to economic development, which has led to numerous environmental problems. It not only threatens the health and prospects of current and future generations, but also undermines the sustainability of long-term growth. While, since 2020, every Chinese individual has basically met their material needs, the government and environmental NGOs have made great efforts to improve residents’ environmental consciousness. Our empirical results indicate that it is of vital necessity for China’s government to strengthen the policy of spreading environmental knowledge and advocating a post-materialistic lifestyle.

There are several limitations to be interpreted carefully as follows: (i) non-random distribution of environmental attributes may lead to endogenous problems, which means that our empirical results only describe the correlation; (ii) our results were generally based on respondents’ self-reported surveys, which means that this article only partially revealed the individuals’ objective environmental attributes; and (iii) last but not least, the year of this survey is another important limitation, which needs to be carefully addressed. Despite these limitations, our research provided a relatively perfect framework to explore the formation of individuals’ PEB, and to discuss the heterogeneous situation of PEB. Accordingly, this study is of vital importance both theoretically and empirically.

## 6. Conclusions

This paper revealed a potential mechanism among PEB, environmental knowledge, environmental perception, and PMV in China. The main contribution of this paper is to construct the SEM including the four factors listed below, simultaneously, and to discuss the structural relationship among them. Meanwhile, the mediating and moderating model was used to discuss the potential mechanism of the generation of Chinese residents’ PEB. Additionally, we discussed the heterogeneity of correlation coefficients between different environmental attributes, so as to enrich the research conclusions.

The important conclusions of this study can be summarized as follows: Firstly, individuals with higher environmental knowledge always show higher passion to PEB. Similar conclusions have been found in the United States, India, New Zealand, and other countries. It indicates that improving environmental knowledge is a feasible way to promote Chinese residents’ PEB. Secondly, environmental perception plays a partially mediating role in the above relationship, but it is not always significant. More specifically, daily environmental perception indirectly impacts the generation of public PEB, whereas ecological environmental perception significantly mediates the formation of private PEB. These conclusions are important, and have not been revealed in previous studies. Thirdly, PMV moderated the formation of PEB systematically. Furthermore, the moderating effect remains positive on each related path. This conclusion indicates that the promotion of PMV is of vital necessity to stimulate Chinese residents’ PEB. It is also consistent with PMV theory proposed by Professor Ingelhart.

This study has a practical value in assessing the formation of Chinese individuals’ PEB. Our results could become the basis for China’s government and environmental NGOs to spread environmental knowledge, advocate a post-materialistic lifestyle, and improve the authenticity of online media reports on environmental issues. More notably, our empirical research provides some meaningful insights for future research works. Environmental perception has been shown to participate in the generation of PEB as a mediator, so other factors affecting PEB (like environmental perception) may also exist, such as government trust or media contact, which may mediate the generation of PEB as well. Future researchers might use the above elements proposed by this study to design a serial multiple-mediator SEM.

## Figures and Tables

**Table 1:** Correlations and descriptive statistics for study variables.

Item					Factor Loading	Mean	SD
Public environmental behavior	Garbage classification				0.544	1.57	0.701
	Rarely use plastic bags				0.609	1.57	0.632
	Pay attention to environmental news				0.546	2.15	0.78
	Discuss environmental issues with others				0.636	1.63	0.700
Private environmental behavior	Donate for environmental protection				0.654	1.20	0.444
	Actively participate in public environmental affairs				0.768	1.27	0.525
	Actively participate in environmental NGOs				0.760	1.19	0.450
	Protect trees or green space spontaneously				0.488	1.19	0.479
	Complaints for environmental protection				0.587	1.11	0.357
Daily environmental knowledge	Overuse of chemical fertilizer is harmful				0.423	0.70	2.504
	Fluorine destroys the ozone layer				0.490	0.79	0.405
	Using coal leads acid rain				0.743	0.44	0.496
Professional Environmental knowledge	Every species is important in the biological chain				0.776	0.47	0.499
	A single species of forest is susceptible to diseases				0.666	0.42	0.493
	Carbon dioxide causes air warming				0.754	0.49	0.500
Daily environmental perception	Air pollution				0.839	3.44	1.888
	Water pollution				0.805	3.51	1.864
	Noise				0.810	3.68	1.906
	Industrial garbage				0.733	4.13	2.057
	Daily garbage				0.671	4.10	1.743
	Lack of green space				0.539	3.60	1.965
	Food pollution				0.705	3.44	2.080
Ecological environmental perception	Forest destruction				0.707	4.65	2.033
	Cultivated land degradation				0.624	4.26	2.084
	Lack of fresh water				0.713	4.29	2.072
	Desertification				0.678	5.44	1.976
	Reduction of wildlife				0.610	4.91	2.177
Post-materialistic value	Government needs democracy				0.597	0.79	0.406
	The people must have a voice of public affairs,				0.735	0.68	0.465
	Representatives should express public opinion,				0.624	0.78	0.417
	People’s benefits are more important than national’s				0.498	0.79	0.404
	People should participate in national decision-making				0.524	0.71	0.453
**Pearson correlation test**	1.	2.	3.	4.	5.	6.	7.
1. Public environmental behavior	1.00						
2. Private environmental behavior	0.23 ***	1.00					
3. Daily environmental knowledge	0.15 ***	0.13 ***	1.00				
4. Professional environmental knowledge	0.19 ***	0.23 ***	0.53 ***	1.00			
5. Daily environmental perception	−0.09 ***	−0.13 ***	−0.18 ***	−0.19 ***	1.00		
6. Ecological environment perception	−0.05 ***	−0.18 ***	−0.04 ***	−0.10 ***	0.56 ***	1.00	
7. Post-materialistic values	0.01	−0.10 ***	0.13 ***	0.09 ***	0.101 ***	0.08 ***	1.00
Means	6.81	5.95	1.99	1.73	25.83	24.23	3.76
SD	0.36	0.02	0.09	0.15	0.13	0.11	0.14

*** significant at *p* < 0.001.

**Table 2:** Mediating coefficients for the explanatory model pathways.

Effects	Daily Knowledge to Public Behavior		Pro-Knowledge to Public Behavior		Daily Knowledge to Private Behavior		Pro-Knowledge to Private Behavior	
	Effect	95%CI	Effect	95%CI	Effect	95%CI	Effect	95%CI
C’	0.496 ***	0.429; 0.563	0.333 ***	0.299; 0.367	0.134 ***	0.074; 0.195	0.165 ***	0133;0.195
b1	−0.031 ***	−0.038; −0.025	−0.029 ***	−0.036; −0.023	−0.002	−0.007; 0.004	0.001	−0.005; 0.006
b2	−0.003	−0.011; 0.005	<0.001	−0.008; 0.008	−0.037 ***	−0.044; −0.030	−0.036 ***	−0.043; −0.029
a1	−2.361 ***	−2.712; −2.009	−1.330 ***	−1.512; −1.147	−2.337 ***	−2.688; −1.985	−1.328 ***	−1.511; −1.145
a2	−0.489 ***	−0.774; −0.204	−0.549 ***	−0.695; −0.398	−0.494 ***	−0.779; −0.208	−0.549 ***	−0.698; −0.401
Effects from environmental knowledge to environmental behavior								
Total	0.571 ***	0.505; 0.637	0.372 ***	0.338; 0.406	0.158 ***	0.097; 0.218	0.184 ***	0.152–0.214
Ind. Total	0.075	0.057–0.095	0.039 ***	0.031; 0.049	0.023 ***	0.007; 0.039	0.019 ***	0.011; 0.027
Ind1(a1 × b1)	0.074 ***	0.054; 0.095	0.039 ***	0.029; 0.050	0.005	−0.009; 0.181	−0.001	−0.008; 0.007
Ind2(a2 × b2)	0.002	−0.003; 0.006	<0.001	−0.004; 0.004	0.018 ***	0.008; 0.030	0.020 ***	0.013; 0.027

*** significant at *p* < 0.001.

**Table 3:** Moderating effects of post-materialistic value on the coefficients.

	Public Environmental Behavior			Private Environmental Behavior		
	β	SE	*p*	β	SE	*p*
Daily environmental knowledge	0.025 ***	0.011	0.019	0.046 ***	0.016	0.005
Professional environmental knowledge	0.024 ***	0.012	0.042	0.036 ***	0.013	0.006
Daily environmental perception	0.029 ***	0.012	0.014	0.053 ***	0.014	0.000
Ecological environment perception	0.024 ***	0.012	0.042	0.036 ***	0.013	0.006

*** significant at *p* < 0.001.

## Data Availability

Data are obtained from the Official website of Chinese General Social Survey. All data permission has been obtained and there is no copyright issue for all the figures in the paper.
